# Effects of Different Occlusal Splints on Joint Vibrations in Bruxers

**DOI:** 10.3390/medicina61061083

**Published:** 2025-06-12

**Authors:** Bora Akat, Ayşe Cavidan Akören, Evşen Tamam

**Affiliations:** 1 Department of Prosthodontics, Faculty of Dentistry, Ankara University, Ankara 06100, Turkey; akoren@ankara.edu.tr; 2 Department of Prosthodontics, Faculty of Dentistry, Gazi University, Ankara 06500, Turkey; evsen78@yahoo.com

**Keywords:** occlusal splint, joint vibration analysis, bruxism

## Abstract

*Background and Objectives*: This study aimed to evaluate the effects of hard, soft, and semi-soft splints on TMJ vibrations in bruxers with JVA and to compare them with data obtained from asymptomatic individuals. *Materials and Methods*: A total of 64 individuals were divided into four subgroups: control (*n* = 15); and hard (*n* = 17), soft (*n* = 16), and semi-soft (*n* = 16) splints. Electrovibratography records from all individuals included in the study before and after the 3-month splint treatment were obtained with the Biopak^®^ System. Joint vibration analysis was used to evaluate TMJ sounds. Data normality was examined with the Kolmogorov–Smirnov and Levene tests. The significance of the differences was investigated by One-Way ANOVA and by the Kruskal–Wallis test. Conover’s multiple comparison test was used in post hoc tests. (ClinicalTrials.gov identifier: NCT06893744, on 24 March 2025, titled; Effects of Different Occlusal Splints). *Results*: After 3 months of treatment, for I < 300 Hz right opening, the control group was statistically lower than both semi-soft (*p* < 0.001) and hard (*p* < 0.001) splint groups. The difference between semi-soft and hard splints in post-treatment I < 300 Hz right opening is not statistically significant. After 3 months of treatment compared with the beginning, the increases in left-opening Ti (*p* = 0.004), I < 300 Hz (*p* = 0.004), and PA (*p* = 0.007) values in the soft splint group were statistically significant. *Conclusions*: All three kinds of splints improved clinical symptoms and complaints of bruxers. For joint vibrations in bruxers, hard and semi-soft splints are more beneficial than soft splints.

## 1. Introduction

Temporomandibular disorders (TMDs) may show signs before symptoms [1]. Being able to recognize these signs and interpret them correctly is an important step in making optimum treatment planning for temporomandibular joint (TMJ).

The concept of TMD, which originates from the occlusal condition, trauma, emotional stress, deep pain input, and parafunction, describes muscle–joint irregularities characterized by symptoms such as pain in the orofacial area, limitation in mouth opening, fatigue in the chewing muscles, and sound in the TMJ [1,2,3]. The most well-known parafunction is bruxism, which can be diurnal or nocturnal and has been shown to be associated with TMD. Okeson [1] states that if parafunctional activity can be controlled, TMD can also be controlled. Dysfunction, one of the common symptoms of functional TMD, is the disruption of the normal condyle–disc relationship with joint sounds [4,5]. Vibration also occurs when joint surfaces rub against each other. It is thought that the higher the roughness of the surfaces, the more vibration occurs. Changes in the joint surface (such as tissue degeneration and disc displacement) can lead to greater friction and vibrations [6]. Healthy TMJ is described as silent [7]. Although one of the most common symptoms of joint dysfunction may be joint sounds/vibrations, it can also be seen in asymptomatic individuals [8,9]. Tallent et al. [10] reported that a clicking sound occurred during mouth opening in 72% of asymptomatic individuals. Although the determination of joint sounds/vibrations is not highly accurate in the diagnosis of TMD, it can be used successfully to compare the differences in joint sounds in symptomatic and asymptomatic individuals [8].

While the intensity of joint vibrations is lower in asymptomatic individuals, the energy of joint vibrations is higher in individuals with TMD. Additionally, there are various waveforms in patients with TMD at different stages [9]. Parafunctions such as bruxism are considered a predisposing factor of TMJ vibrations. It has been suggested that erosions occur on the joint surfaces in individuals with bruxism as a result of the increased friction between the joint surfaces, and this creates low-intensity vibrations [11].

Joint vibration analysis (JVA) based on spectral analysis can be used to examine and record joint sounds. Providing fast, simple, and repeatable measurements, JVA records TMJ vibrations. It enables the identification of vibrations during opening and closing movements. JVA, a non-invasive technique, is a useful method for diagnosing TMD [9]. TMJ vibration analysis measures the intensity and frequency of vibrations emanating from the joint throughout all movements of the jaw. Vibrations can be recorded below one micrometer [6].

No known treatment method can cure bruxism permanently. Occlusal splints with different features are used to control bruxism, relieve symptoms, and prevent further injuries. This study aimed to evaluate the effects of hard, soft, and semi-soft splints used in the treatment of bruxism on TMJ vibrations with JVA and compare them with data obtained from asymptomatic individuals. The hypothesis of this study is that the joint vibrations following the wear of soft occlusal splints may be higher than those with semi-soft and hard occlusal splints.

## 2. Materials and Methods

**Figure 1:**
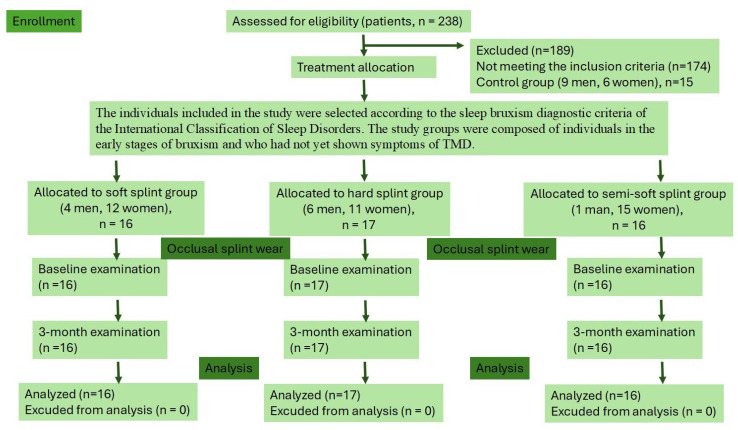
CONSORT flow diagram. Of the 238 individuals assessed for eligibility, 174 were excluded based on the excluding criteria given in Supplementary Materials (File S1).

In total, 238 individuals were single-arm examined (BA) for study eligibility between March 2013 and May 2014 in the Department of Prosthodontics, Faculty of Dentistry, Ankara University in Ankara, Türkiye. A total of 64 individuals (49 with bruxism and 15 healthy; 20 men and 44 women, aged 18 to 42) were included in this controlled clinical study. The study protocol was approved by the Clinical Research Ethics Committee (decision #42, 17 December 2012) of Ankara University Faculty of Dentistry, and carried out in accordance with the CONSORT guidelines (Figure 1). The study was performed in accordance with the Helsinki Declaration of 1975 (ClinicalTrials.gov identifier: NCT06893744, on 24 March 2025, titled “Effects of Different Occlusal Splints”). All the study participants obtained written informed consent following clinical examinations.

### 2.1. Study Groups

The individuals included in the study were selected according to the sleep bruxism diagnostic criteria of the International Classification of Sleep Disorders [12]. A priori power analysis was conducted using G*Power (Version 3.1.9.7, Universität Düsseldorf, Düsseldorf, Germany) to determine the required sample size for detecting a medium effect size (f = 0.25) at a significance level of 0.05 with 80% power in a One-Way ANOVA design with four groups. The analysis indicated that a total sample size of 60 participants was required. Accordingly, 64 individuals were included in this study.

The study groups were composed of individuals in the early stages of bruxism and who had not yet shown symptoms of TMD. The control group was composed of healthy individuals, and those with any of the conditions listed as criteria for inclusion (Table 1) in the study group were not included.

Participants diagnosed with bruxism were randomly assigned to one of three intervention groups (soft splint, hard splint, or semi-soft splint) using a computer-generated randomization sequence created via randomizer.org. Allocation was carried out by an independent researcher who was not involved in clinical procedures or outcome evaluation.

Due to the physical characteristics of the splints, blinding of participants and clinicians was not feasible. However, the clinicians who performed the outcome assessments were blinded to group allocation to reduce assessment bias.

The patients in the study group were randomly divided into subgroups, namely hard, soft, and semi-soft, in terms of the occlusal splints to be used. The control group consisted of asymptomatic individuals (9 men and 6 women, mean age 21.08, aged between 19 and 25). Study groups were formed as soft splint (SS) group (*n* = 16, 4 men and 12 women, mean age 22.06, aged between 19 and 24), hard splint (HS) group (*n* = 17, 6 men and 11 women, mean age 22.05, aged between 20 and 31), and semi-soft splint (SSS) group (*n* = 16, 1 man and 15 women, mean age 23.62, aged between 19 and 42).

### 2.2. Clinical Examinations

Anamnesis was recorded (parafunctional habits were assessed), and clinical examinations (dental, TMJ, and masticatory muscles) were performed on all included individuals at baseline and after 3 months of splint usage (Supplementary Files S2 and S3).

### 2.3. JVA Records

Electrovibratography (EVG) records from all individuals included in the study before treatment and following 3-month splint treatment were recorded with the Biopak^®^ System (Version 5.3i, BioResearch Inc., Milwaukee, WI, USA). JVA was used to evaluate TMJ sounds. During recording, individuals were seated in a stable position, with their backs upright and their feet on the ground. Two sensors, consisting of piezoelectric accelerometers placed on the right and left ends of an adjustable headset, were placed in the lateral poles of the TMJ condyles, and the red (right) and blue (left) cables from the sensors were connected to the amplifier. Then, the individual’s maximum mouth-opening amount was determined as directed by the program. With the mouth in the widest possible position, the distance between the incisal edges of the upper and lower central teeth was measured and added to the overbite amount and recorded in the BioPAK^®^ program. BioPAK^®^ program gives the maximum mouth opening as the sum of the distance between the upper and lower central teeth and the amount of vertical overlap. Individuals repeated jaw movements in synchrony with the metronome on the computer screen. These jaw movements involve opening the mouth as wide as possible and then closing the mouth so that the sound of teeth clashing can be heard. Each on–off cycle takes 1.5 s. The individual was recorded by repeating jaw movements 10 times (10 cycles) in coordination with a metronome. These EVG recordings obtained at X5 amplification were examined in the time–frequency graph. With the BioPAK^®^ program, the numerical values of each selected vibration region and the average of the values of the six regions were obtained for the total integral (Ti), integral above 300 Hz (I > 300 Hz), integral below 300 Hz (I < 300 Hz), I > 300 Hz/I < 300 Hz ratio, peak amplitude (PA), peak frequency (PF), and median frequency (MF) parameters.

### 2.4. Occlusal Splint Treatment

**Figure 2:**
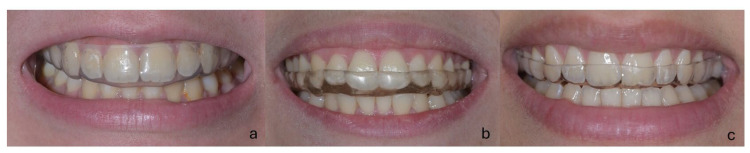
Different types of occlusal splints: (**a**) hard, (**b**) soft, and (**c**) semi-soft.

In the study, individuals in the control group were not allowed to use any occlusal appliance, but JVA records were repeated after 3 months. Among the bruxism patients participating in the study, a soft occlusal splint (BIOPLAST^®^, Erkrath, Germany) was used in the SS group, a hard occlusal splint produced individually using self-cured acrylic resin (DURA SPLINT^®^, Gold Coast, Australia) was used in the HS group, and a semi-soft occlusal splint (DURASOFT^®^ pd, Iserlohn, Germany) was used in the SSS group for 3 months (Figure 2).

### 2.5. Statistics

All the data obtained in the study were analyzed statistically using the IBM SPSS Statistics 17.0 (IBM Corporation, Armonk, NY, USA) package program. The normality of the distribution of the variables was examined with the Kolmogorov–Smirnov test, and the homogeneity of the variances with the Levene test. The significance of the difference between the groups in terms of means was investigated by One-Way ANOVA, and in terms of medians, it was investigated by the Kruskal–Wallis test. Conover’s multiple comparison test was used in post hoc tests. The significance of the differences between pre-treatment and post-treatment, right side and left side, and opening position and closing position within the groups was evaluated using the Wilcoxon Sign test. Categorical variables were examined with Pearson’s Chi-Square or Fisher’s Exact Probability test. Statistically significant correlations between continuous variables, if any, were investigated with Spearman’s Correlation Test. Unless stated otherwise, results for *p* < 0.05 were considered statistically significant. However, Bonferroni Correction was used to control Type I errors in all possible multiple comparisons.

## 3. Results

**Figure 3:**
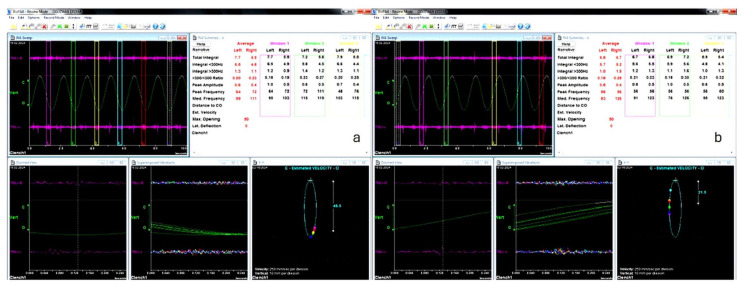
Measured JVA parameters: (**a**) opening movement and (**b**) closing movement.

Anamnesis and clinical examination evaluations for all three groups before and after treatment are given in the Supplementary Tables (Supplementary Files S2 and S3). The values of the measured JVA parameters for all groups in opening and closing movements, for the right and left joints, initially and after treatment are shown in the graphs (Figure 3).

When SS, HS, SSS, and control groups were compared in terms of initial recordings, there was no statistical difference in direction and movement type for Ti, I > 300 Hz, I < 300 Hz, ratio, PA, PF, and MF (Table 2).

When SS, HS, SSS, and control groups were compared in terms of 3-month post-treatment recordings, I < 300 Hz right-opening direction showed a statistical difference (*p* = 0.006) (Table 3).

In the post-treatment recordings for I < 300 Hz right opening, the control group was statistically lower than both SS (*p* < 0.001) and HS (*p* < 0.001). The difference between SS and HS in post-treatment I < 300 Hz right opening is not statistically significant (Table 4).

When SS, HS, SSS, and control groups were compared in terms of measurement time (baseline and post-treatment), the increases in left-opening Ti (*p* = 0.004), I < 300 Hz (*p* = 0.004), and PA (*p* = 0.007) values in the SS group were statistically significant (Table 5; Supplementary File S4).

## 4. Discussion

Although the number of studies on the treatment of bruxism is increasing day by day, debates still continue on which of the hard or soft occlusal splints are more beneficial. For this reason, decisions of which appliance or combination of appliances will be used when treating bruxism are made based on experiences [13]. Regarding bruxism and its effects, TMJ vibrations may be a factor to be evaluated in making the diagnosis. It has been suggested that in individuals with a habit of clenching their teeth, the friction between the joint surfaces increases, causing erosion and creating low-intensity vibrations [11].

Hwang et al. [14] stated that the high ratio of frequency values above 300 Hz to frequency values below 300 Hz is related to the advanced level of degeneration in the joint. Ishigaki et al. [15] stated that a low-frequency (below 300 Hz) clicking sound is encountered in reduced disc displacement, and high-frequency (above 300 Hz) crepitus is encountered in degenerative cases.

Soft splints have disadvantages such as not fully stabilizing the TMJ, not being suitable for occlusal addition, being difficult to polish, easily wearing out, and allowing limited occlusal corrections. For these reasons, our study set out with the hypothesis that joint vibrations in patients using soft occlusal splints may be higher than those in patients using semi-soft and hard occlusal splints. Patients with bruxism were divided into three groups, and a different occlusal splint (hard, soft, and semi-soft) was worn for 3 months in each group. Each patient was clinically evaluated before, and at the end of occlusal splint treatment, an anamnesis form was filled out in order to question their complaints about bruxism, and joint vibrations were recorded with the JVA device. Patients in the control group were selected from asymptomatic individuals who did not show signs of bruxism. In the evaluations, improvements were observed in clinical evaluations and complaints related to bruxism in patients wearing occlusal splints. In terms of joint vibrations, it was observed that the values in the control group and in patients using hard and semi-soft splints were lower than those using soft splints.

Symptoms of bruxism include tooth wear, tooth mobility, pain, and hypertrophy in the masticatory muscles, fractures and decementation in restorations, abfraction, externally audible teeth grinding, a feeling of tiredness in the jaws, and bite marks on the tongue and cheeks [1,16,17,18]. The diagnostic criteria of the patients with bruxism included in our study are consistent with the literature. In the pre-treatment clinical examination, 48.97% of the 49 patients with bruxism had tooth wear, 63.26% had masseter hypertrophy, 61.22% had tooth sensitivity, 32.65% had tongue and/or cheek marks, 6.12% had abfraction, 6.12% had tooth mobility, 22.44% had fractures in teeth and/or restorations, 24.48% had pain in the TMJ, and 95.91% had pain in the masticatory muscles (Supplementary File S2). According to the anamnesis forms of the 49 patients before the treatment, 55.10% reported that someone heard them grinding their teeth at night, and 65.30% during the day, and 89.79% reported a feeling of tiredness in the jaw when they woke up (Supplementary File S3).

Singh et al. [19] stated that there are many treatment options, such as occlusal splints, emotional stress therapy, physiotherapy, and pharmacological treatment in treating bruxism, but the most popular among these is occlusal splint treatment. In our study, asymptomatic individuals constituted the control group. Forty-nine patients with bruxism were randomly divided into three groups, and soft, hard, and semi-soft splints were used separately for each group. Dube et al. [20] reported that wearing an occlusal splint reduces the frequency of bruxism and muscle activity in patients with sleep bruxism. In a study [21] comparing hard and soft splints, it was reported that both types of splints provided a decrease in clinical symptoms, and a statistical decrease in occlusal forces was also detected with the soft splint. Klasser et al. [22] also stated that the hard splint was more successful than the soft splint regarding muscle activities. In our study, in line with the literature, in the soft splint group, 12 patients had pain during palpation of the masseter muscle before treatment, while 7 patients had pain after treatment. In the hard splint group, masseter pain in 14 patients before treatment was relieved in 11 patients after treatment. In the semi-soft splint group, masseter pain in 14 patients before treatment was relieved in 9 patients after treatment. When the anamnesis forms were evaluated, it was observed that the patients’ waking pain improved at various rates after the use of all three splint types (Supplementary File S2). Within the current study’s limitations, it was thought that the improvements in masseter pain were better in those using hard splints and semi-soft splints than in those using soft splints.

The clinical differences observed between splint types may be attributed to their distinct biomechanical and neurophysiological effects on the masticatory system. Hard splints are known to provide greater occlusal stability and may help distribute occlusal forces more evenly, thereby reducing parafunctional muscle activity [1]. They can also diminish proprioceptive feedback from the periodontal ligaments, potentially modulating neuromuscular activity patterns [23]. In contrast, soft splints may offer improved patient comfort but exert their influence primarily through cushioning effects rather than through stabilization. Some studies suggest that their resilience may actually lead to increased muscle activity due to a rebound phenomenon, particularly during sleep bruxism episodes [24]. Semi-soft splints aim to combine the mechanical stability of hard splints with the comfort of soft ones; however, the clinical outcomes associated with this design are still not well-established and likely influenced by individual variation in neuromuscular adaptation.

Recent studies have investigated the biomechanical and neuromuscular effects of various occlusal splint types in patients with bruxism. A study by Akat et al. [25] demonstrated that all three types of splints—hard, soft, and semi-soft—significantly reduced electromyographic (EMG) activity of masticatory muscles after three months of use, with the most pronounced decrease observed in the hard splint group. This suggests that hard splints may provide greater occlusal stability, leading to more effective muscle relaxation. In contrast, soft splints, while offering increased comfort, may not provide the same level of muscular inhibition. A study by Zieliński et al. [26] found that soft stabilization splints did not significantly reduce jaw muscle activity compared to baseline values. This indicates that while soft splints may be more comfortable, they might be less effective in reducing muscle hyperactivity associated with bruxism.

There is a consensus that limitation in maximum mouth opening is a symptom that may occur in TMJ-related problems [1]. In our study, the mean maximum mouth opening of asymptomatic individuals in the control group (52.33 mm) was found to be higher than that of bruxism patients in the study group (47.95 mm). Various types of splint treatments have been shown in the literature to increase maximum mouth opening [27] statistically. In our study, it was observed that more patients had an increase in maximum mouth opening with the semi-soft splint than with the hard and soft splints (Supplementary File S3).

JVA records and analyzes joint vibrations, and the total integral (Ti), that is, the intensity or loudness of the sound, can be determined. Ti expresses the intensity of the JVA signal at all frequencies. The ratio is the division of the analog signal intensities above and below 300 Hz and indicates soft and hard tissue pathologies. Peak frequency expresses the largest frequency in the frequency spectrum and is the region where JVA vibrations are most intense. Peak amplitude is the absolute amplitude of the peak frequency. Median frequency is the frequency at which the frequency spectrum is divided into two regions with equal power [9,28]. In our study, all the mentioned parameters of JVA were evaluated for all groups, both before and after treatment.

Bruxism may cause joint vibrations and/or joint sounds [11,29]. Guler et al. [4] examined patients with TMD accompanied by bruxism and those without bruxism and reported that statistically significant joint sounds were detected in patients with bruxism and reduced disc displacement. In addition, it has been stated that joint vibrations and/or joint sounds may also occur in asymptomatic individuals and that no problems develop in patients even if they are not treated [8,9,29]. It has been reported that the I > 300 Hz/I < 300 Hz (ratio) values in asymptomatic individuals are less than 0.5 [28], and in another study [15], it is between 0.02 and 0.30. In our study, the average ratio value in asymptomatic individuals was 0.53. This finding supports the idea that joint vibrations can also be seen in asymptomatic individuals.

Hwang et al. [14] recorded the joint vibrations of symptomatic and asymptomatic individuals with clicking sounds and muscle pain in both joints, using JVA, and reported that the ratio values differed for both groups. The ratio values in symptomatic patients varied between 0.08 and 0.44. Our study determined the average ratio as 0.47 in patients with bruxism. It has been reported that vibrations below 300 Hz are high in patients with a clicking sound accompanied by reduced disc displacement [15]. For this reason, it is expected that the ratio values will be lower in individuals with clicking sounds. The slightly higher ratio in our study may be related to the absence of clicking sounds in most individuals with bruxism.

It has been reported that the total integral and peak amplitude values during opening/closing movements of individuals with clicking sound and muscle pain are statistically higher than those of asymptomatic individuals. It has been reported that vibrations are seen at frequencies above 50 Hz in symptomatic individuals and that the frequency at which vibrations are recorded decreases to below 15 Hz following treatment [30]. Sano et al. [8] reported higher amplitudes in individuals with TMD symptoms compared to asymptomatic individuals. In our study, when the first and second recordings within the groups were compared, the increase in the SS group and the decrease in the SSS group for some parameters (I < 300 Hz opening left, PA opening left) were found to be significant. This situation can be attributed to the increase in the undesired forces causing joint vibrations in the soft splint group and the decrease in these forces in those wearing semi-soft splints.

Christensen and Orloff [31] performed joint vibration analysis three times with 3-min intervals on TMD and asymptomatic individuals and stated that statistical repeatability was demonstrated between measurements. Zhang et al. [28] examined the repeatability of vibration measurements on 34 asymptomatic individuals and reported that there were no changes in measurements repeated with 3-min intervals, but there were statistically significant differences in total integral and peak amplitude values in measurements made 1 week later. In the study, the reference peak frequency for asymptomatic individuals was reported to be between 13 and 126 Hz, and the median frequency was between 60 and 232 Hz. In our study, the average peak frequency of individuals in the control group was determined to be 86.63 Hz, and the average median frequency was determined to be approximately 210.85 Hz, which is consistent with the reference values mentioned.

Joint vibrations of individuals with and without bruxism were compared, and no difference was found between patients with mild bruxism and asymptomatic individuals in terms of JVA parameters. In the group with more severe bruxism, total integral, I < 300 Hz, I > 300 Hz, peak amplitude, and peak frequency were found to be statistically higher than in asymptomatic individuals. However, in the more severe bruxism group, total integral, I < 300 Hz, I > 300 Hz, and peak amplitude were found to be statistically higher than in the mild bruxism group [9]. It has been stated that peak frequency, median frequency, peak amplitude, ratio, and total integral were higher in the TMD group than in asymptomatic individuals [32]. Our study observed that the values of patients with bruxism for the parameters of total integral, I > 300 Hz, I < 300 Hz, peak amplitude, and peak frequency are higher than the values of asymptomatic individuals. For patients with bruxism, the mean Ti was 5.67 PaHz, while it was 4.4 PaHz for asymptomatic individuals. The mean I < 300 Hz was 3.94 PaHz with bruxism; for the control, it was 2.93 PaHz. For the patients with bruxism, the mean I > 300 Hz was 1.58 PaHz, while it was 1.47 PaHz for the control. The mean PA was 0.29 Pa for the bruxism groups; for asymptomatics, it was 0.20 Pa. As for the mean PF, 110.70 Hz and 86.71 Hz were the values determined for working and control groups, respectively.

Wearing an occlusal splint has been shown to reduce joint vibrations and/or joint sounds generally. Turcio et al. [30] reported a statistically significant decrease in joint vibrations after treatment in patients who underwent exercise and splint therapy. Conti et al. [31] found a decrease in joint sounds with the anterior positioning splint and an increase with NTI-tss after 3 months of wearing. Mazetto et al. [33] also stated that wearing a stabilization splint and an anterior positioning splint caused a decrease in total integral (Ti) values. In our study, when the records taken at the beginning and following 3 months of splint treatment were examined, the increase in the SS group was found to be statistically significant for Ti-opening left. The decreases between the initial and post-treatment recordings of the HS and SSS groups for Ti-opening left are statistically insignificant. The increase in the SS group in terms of Ti value may indicate that the amount of energy in the joint has increased; therefore, the expected improvement in joint vibrations from the occlusal splint has not occurred, while the decrease in the HS and SS groups may indicate that improvement has occurred. However, although not statistically significant, an increase was observed after treatment in the SS group for Ti-closure left compared to the beginning. Similarly, when the initial and post-treatment recordings were compared for PA opening left and I < 300 Hz opening left, the increase in the SS group was found to be statistically significant (Table 4). When splint types are compared, the values obtained with the soft splint for PA-opening left are statistically different from the other two splints’ values. The differences between the values obtained with soft and semi-soft splints for Ti-opening left, and I < 300 Hz-opening left are statistically significant (Table 4). Although no participant fulfilled the diagnostic criteria for clinically relevant TMD, JVA data may reflect early-stage or subclinical intra-articular alterations. The reduction of joint vibrations following splint use was interpreted as a favorable sign, potentially indicating improved joint coordination or reduction in condylar distraction. Different splint materials may distribute occlusal forces differently and affect joint loading patterns, which in turn may lead to distinct effects on the disc–condyle relationship and joint vibrations.

The findings of this study offer clinically relevant insights for the management of patients with early-stage bruxism. The observed differences in joint sound attenuation and muscle activity patterns suggest that splint-material selection may influence treatment outcomes. For instance, semi-soft splints may be preferable for patients requiring both occlusal stabilization and muscle relaxation, while hard splints might be more appropriate when greater vertical dimension control is needed. Furthermore, the reduction in joint vibrations associated with certain splint types may indicate improved disc–condyle coordination, supporting their use in preventive or early-intervention strategies to avoid progression to symptomatic TMD.

This study has several limitations that must be acknowledged. First, the sample size was relatively small, which may limit the statistical power and generalizability of the findings. Second, there was a notable gender imbalance among the groups, particularly in the semi-soft splint group, which consisted predominantly of female participants. Third, the follow-up period was limited to 3 months; while short-term effects of occlusal splints could be observed, long-term outcomes remain unclear. Finally, no data were collected regarding the sustainability of treatment effects or patient compliance beyond the study period. Future research should consider larger, more balanced cohorts and extended follow-up durations to validate and expand upon these findings.

One other limitation of the present study is the partial implementation of blinding. Due to the physical characteristics and visible differences of the occlusal splints used (soft, hard, and semi-soft), it was not feasible to blind participants or clinicians during the treatment phase. Although outcome assessors were blinded to the group allocation to minimize evaluation bias, the absence of full blinding may have introduced some risk of performance bias. Future studies could consider incorporating double-blind protocols where possible, or using alternative methods to further reduce the risk of bias.

A further limitation of the present study is the unequal gender distribution across groups, with a predominance of female participants. While this reflects the demographic characteristics of the clinical population at the time of recruitment, it may limit the generalizability of the findings, especially in terms of potential sex-related differences in splint efficacy or bruxism patterns. Additionally, although participants’ age data were recorded and comparable among groups, other socio-demographic variables, such as educational background, employment status, and income level, were not collected. These factors can influence oral health behaviors, stress levels, and treatment adherence, and may therefore have an indirect effect on bruxism severity and splint efficacy. The absence of such data limits the ability to fully interpret the generalizability of the results. Future studies should aim to include a more balanced gender representation to confirm these findings across sexes.

## 5. Conclusions

Within the limitations of the present study, the following conclusions were drawn:

(1) All three occlusal splints improved clinical symptoms and complaints of individuals with bruxism.
(2) The total energy of vibrations in the joint (Ti) decreases with the use of hard and semi-soft splints, while its increase with soft splints is significant.
(3) It was observed that all three splints tested could be used in patients with bruxism, but hard and semi-soft splints were more beneficial in terms of joint vibrations.
(4) These results support the clinical consideration of semi-soft splints as a potentially superior alternative for patients with early-stage bruxism, offering a balance between flexibility and support to preserve joint health.

## Figures and Tables

**Table 1:** Criteria for inclusion.

Sleep Bruxism Diagnostic Criteria of the International Classification of Sleep Disorders
A. The patient has a complaint of grinding/clenching during sleep.
B. One or more of the following occurs:
- Abnormal wear of the teeth;
- Sounds associated with the bruxism;
- Jaw muscle discomfort.
C. No other medical or mental disorders that affect masticatory muscles activity.

**Table 2:** Comparison among the groups in terms of initial recording.

Variables	Groups	N	Mean	Std. Deviation	Median	Minimum	Maximum	*p*-Value *
**Tİ opening left**	*Soft*	16	5.06	2.07	4.55	2.60	10.00	0.141
	*Hard*	17	6.85	4.49	5.50	3.20	21.70	
	*Durasoft*	16	6.97	4.27	5.15	3.20	16.70	
	*Control*	15	4.59	1.01	4.40	3.10	6.70	
**Tİ opening right**	*Soft*	16	5.53	1.66	5.50	3.50	10.70	0.179
	*Hard*	17	6.82	3.25	6.20	3.40	15.90	
	*Durasoft*	16	6.78	3.59	5.45	4.10	16.10	
	*Control*	15	4.81	0.80	4.90	3.10	5.90	
**Tİ closing left**	*Soft*	16	5.10	1.82	4.70	2.60	8.70	0.535
	*Hard*	17	5.71	3.18	5.10	2.70	15.40	
	*Durasoft*	16	4.94	1.67	4.45	3.30	8.40	
	*Control*	15	4.38	0.91	4.20	3.20	6.80	
**Tİ closing right**	*Soft*	16	5.46	1.34	5.35	3.30	8.10	0.470
	*Hard*	17	5.48	2.02	4.90	3.40	10.60	
	*Durasoft*	16	4.98	1.06	4.80	3.80	7.40	
	*Control*	15	4.73	0.80	5.00	3.20	6.00	
**I < 300 opening left**	*Soft*	16	3.61	2.03	3.00	1.80	8.70	0.175
	*Hard*	17	4.94	3.52	3.80	1.80	15.50	
	*Durasoft*	16	5.26	3.74	3.75	1.90	13.70	
	*Control*	15	3.13	0.72	3.10	1.70	4.70	
**I < 300 opening right**	*Soft*	16	3.79	1.68	3.50	2.10	9.20	0.137
	*Hard*	17	5.02	2.94	4.30	2.30	13.90	
	*Durasoft*	16	4.84	2.87	3.75	2.40	12.30	
	*Control*	15	3.20	0.83	3.20	1.50	4.80	
**I < 300 closing left**	*Soft*	16	3.62	1.77	3.15	1.60	7.20	0.854
	*Hard*	17	4.09	2.53	3.40	1.70	10.60	
	*Durasoft*	16	3.56	1.66	3.15	1.80	7.00	
	*Control*	15	3.02	0.83	3.10	1.70	4.80	
**I < 300 closing right**	*Soft*	16	3.66	1.36	3.30	1.90	6.40	0.717
	*Hard*	17	3.84	1.63	3.10	2.20	8.60	
	*Durasoft*	16	3.34	1.27	3.10	2.00	6.40	
	*Control*	15	3.17	0.90	3.00	1.50	4.50	
**I > 300 opening left**	*Soft*	16	1.44	0.38	1.45	0.80	2.00	0.433
	*Hard*	17	1.90	1.22	1.60	0.90	6.20	
	*Durasoft*	16	1.71	0.57	1.50	1.00	3.00	
	*Control*	15	1.44	0.51	1.40	0.70	2.20	
**I > 300 opening right**	*Soft*	16	1.74	0.46	1.55	1.00	2.60	0.813
	*Hard*	17	1.79	0.69	1.80	1.00	4.00	
	*Durasoft*	16	1.95	1.01	1.70	1.10	5.40	
	*Control*	15	1.61	0.59	1.60	0.70	2.50	
**I > 300 closing left**	*Soft*	16	1.48	0.34	1.45	0.90	2.00	0.795
	*Hard*	17	1.61	0.90	1.40	0.90	4.70	
	*Durasoft*	16	1.39	0.33	1.40	0.80	2.00	
	*Control*	15	1.35	0.47	1.40	0.70	2.10	
**I > 300 closing right**	*Soft*	16	1.80	0.43	1.75	1.10	2.60	0.376
	*Hard*	17	1.62	0.73	1.50	0.90	4.10	
	*Durasoft*	16	1.61	0.44	1.70	0.90	2.50	
	*Control*	15	1.59	0.59	1.50	0.70	2.70	
**Ratio opening left**	*Soft*	16	0.48	0.24	0.43	0.15	1.05	0.789
	*Hard*	17	0.45	0.20	0.41	0.11	0.87	
	*Durasoft*	16	0.41	0.16	0.37	0.21	0.67	
	*Control*	15	0.48	0.19	0.44	0.21	0.84	
**Ratio opening right**	*Soft*	16	0.52	0.25	0.47	0.17	1.21	0.429
	*Hard*	17	0.41	0.17	0.39	0.14	0.70	
	*Durasoft*	16	0.45	0.20	0.43	0.19	0.99	
	*Control*	15	0.56	0.29	0.58	0.17	1.08	
**Ratio closing left**	*Soft*	16	0.50	0.26	0.46	0.21	1.21	0.969
	*Hard*	17	0.46	0.23	0.38	0.17	0.93	
	*Durasoft*	16	0.47	0.22	0.45	0.18	0.88	
	*Control*	15	0.49	0.25	0.41	0.20	0.87	
**Ratio closing right**	*Soft*	16	0.56	0.28	0.48	0.25	1.34	0.732
	*Hard*	17	0.45	0.16	0.46	0.22	0.77	
	*Durasoft*	16	0.55	0.28	0.52	0.17	1.07	
	*Control*	15	0.57	0.30	0.59	0.17	1.14	
**PA opening left**	*Soft*	16	0.28	0.26	0.20	0.10	1.00	0.200
	*Hard*	17	0.40	0.38	0.30	0.10	1.60	
	*Durasoft*	16	0.35	0.28	0.30	0.10	1.10	
	*Control*	15	0.21	0.07	0.20	0.10	0.30	
**PA opening right**	*Soft*	16	0.24	0.17	0.20	0.10	0.80	0.130
	*Hard*	17	0.36	0.27	0.30	0.10	1.10	
	*Durasoft*	16	0.33	0.19	0.30	0.10	0.70	
	*Control*	15	0.21	0.06	0.20	0.10	0.30	
**PA closing left**	*Soft*	16	0.30	0.22	0.25	0.10	0.80	0.907
	*Hard*	17	0.31	0.19	0.30	0.10	0.70	
	*Durasoft*	16	0.29	0.18	0.20	0.10	0.70	
	*Control*	15	0.24	0.08	0.20	0.10	0.40	
**PA closing right**	*Soft*	16	0.30	0.19	0.20	0.10	0.80	0.529
	*Hard*	17	0.29	0.11	0.30	0.10	0.50	
	*Durasoft*	16	0.30	0.18	0.30	0.10	0.80	
	*Control*	15	0.23	0.09	0.20	0.10	0.40	
**PF opening left**	*Soft*	16	129.44	123.92	78.00	25.00	447.00	1.000
	*Hard*	17	122.41	112.82	72.00	29.00	357.00	
	*Durasoft*	16	121.38	143.02	77.50	25.00	498.00	
	*Control*	15	95.73	76.70	72.00	41.00	326.00	
**PF opening right**	*Soft*	16	143.81	108.66	97.00	33.00	396.00	0.297
	*Hard*	17	108.29	103.40	60.00	33.00	357.00	
	*Durasoft*	16	117.75	124.99	76.00	37.00	498.00	
	*Control*	15	97.27	102.24	64.00	29.00	423.00	
**PF closing left**	*Soft*	16	85.75	105.53	42.50	25.00	384.00	0.489
	*Hard*	17	101.18	115.48	56.00	29.00	439.00	
	*Durasoft*	16	150.06	179.24	58.00	17.00	482.00	
	*Control*	15	99.73	129.70	52.00	33.00	490.00	
**PF closing right**	*Soft*	16	107.94	119.60	48.00	29.00	439.00	0.869
	*Hard*	17	78.06	80.09	44.00	25.00	330.00	
	*Durasoft*	16	165.63	194.18	42.50	17.00	482.00	
	*Control*	15	98.00	130.72	52.00	33.00	494.00	
**MF opening left**	*Soft*	16	196.19	60.34	191.00	91.00	306.00	0.912
	*Hard*	17	196.41	55.04	193.00	68.00	283.00	
	*Durasoft*	16	186.50	39.09	187.00	123.00	244.00	
	*Control*	15	195.53	41.52	185.00	123.00	267.00	
**MF opening right**	*Soft*	16	213.44	56.70	220.00	115.00	322.00	0.385
	*Hard*	17	190.59	48.93	189.00	107.00	255.00	
	*Durasoft*	16	193.63	44.54	185.00	130.00	298.00	
	*Control*	15	218.27	59.28	216.00	119.00	314.00	
**MF closing left**	*Soft*	16	193.25	67.21	192.50	91.00	318.00	0.915
	*Hard*	17	188.47	63.09	173.00	115.00	287.00	
	*Durasoft*	16	186.88	63.15	191.00	95.00	275.00	
	*Control*	15	195.93	59.05	169.00	119.00	279.00	
**MF closing right**	*Soft*	16	212.19	64.57	210.50	83.00	330.00	0.691
	*Hard*	17	193.29	40.39	181.00	134.00	267.00	
	*Durasoft*	16	203.31	66.46	202.50	91.00	306.00	
	*Control*	15	217.27	63.93	224.00	123.00	318.00	

* According to Kruskal Wallis test, Bonferroni Correction, results were considered statistically significant for *p* < 0.0063.

**Table 3:** Comparison among the groups in terms of 3-month post-treatment recordings.

Variables	Groups	N	Mean	Std. Deviation	Median	Minimum	Maximum	*p*-Value *
**Tİ opening left**	*Soft*	16	6.08	3.28	4.90	3.40	15.90	0.139
	*Hard*	17	6.29	4.14	4.30	3.00	15.20	
	*Durasoft*	16	5.01	2.43	4.10	3.30	12.90	
	*Control*	15	4.25	1.12	3.80	3.00	7.20	
**Tİ opening right**	*Soft*	16	5.56	1.26	5.40	3.90	8.10	0.008
	*Hard*	17	6.28	2.97	5.30	3.80	16.10	
	*Durasoft*	16	5.74	2.59	4.90	3.50	12.70	
	*Control*	15	4.27	0.54	4.10	3.30	5.30	
**Tİ closing left**	*Soft*	16	4.96	1.47	4.80	2.90	8.40	0.094
	*Hard*	17	4.85	2.58	4.20	2.70	13.70	
	*Durasoft*	16	3.96	0.68	4.05	2.90	5.40	
	*Control*	15	3.87	0.52	3.80	3.20	4.70	
**Tİ closing right**	*Soft*	16	5.23	1.48	5.00	3.50	9.20	0.253
	*Hard*	17	4.69	1.01	4.70	3.20	7.50	
	*Durasoft*	16	4.46	0.80	4.40	3.10	5.80	
	*Control*	15	4.31	0.59	4.20	3.50	5.40	
**I < 300 opening left**	*Soft*	16	4.62	3.24	3.55	2.20	14.50	0.106
	*Hard*	17	4.91	3.86	3.30	1.70	13.30	
	*Durasoft*	16	3.48	2.07	2.80	1.90	9.90	
	*Control*	15	2.91	1.10	2.80	1.80	5.60	
**I < 300 opening right**	*Soft*	16	3.93	1.22	4.00	2.20	6.60	0.006
	*Hard*	17	4.65	2.65	3.90	2.30	13.40	
	*Durasoft*	16	3.96	2.38	2.90	1.80	10.80	
	*Control*	15	2.75	0.65	2.60	1.60	4.00	
**I < 300 closing left**	*Soft*	16	3.58	1.39	3.45	1.90	7.10	0.023
	*Hard*	17	3.49	2.33	2.90	1.40	11.60	
	*Durasoft*	16	2.55	0.68	2.45	1.60	3.70	
	*Control*	15	2.51	0.35	2.50	1.90	3.00	
**I < 300 closing right**	*Soft*	16	3.64	1.36	3.50	2.00	7.50	0.117
	*Hard*	17	3.25	1.07	3.20	1.70	6.20	
	*Durasoft*	16	2.81	0.81	2.60	1.60	4.60	
	*Control*	15	2.82	0.52	2.80	1.90	3.70	
**I > 300 opening left**	*Soft*	16	1.48	0.34	1.40	1.00	2.30	0.648
	*Hard*	17	1.37	0.39	1.40	0.80	2.10	
	*Durasoft*	16	1.54	0.51	1.50	1.00	3.10	
	*Control*	15	1.34	0.26	1.30	0.90	1.70	
**I > 300 opening right**	*Soft*	16	1.63	0.31	1.70	1.00	2.20	0.360
	*Hard*	17	1.61	0.48	1.60	0.80	2.70	
	*Durasoft*	16	1.78	0.48	1.75	1.00	2.70	
	*Control*	15	1.52	0.29	1.50	0.90	1.90	
**I > 300 closing left**	*Soft*	16	1.31	0.30	1.25	0.90	1.90	0.725
	*Hard*	17	1.34	0.40	1.30	0.70	2.20	
	*Durasoft*	16	1.42	0.31	1.40	1.00	2.20	
	*Control*	15	1.36	0.31	1.30	0.90	2.00	
**I > 300 closing right**	*Soft*	16	1.56	0.33	1.50	1.10	2.20	0.496
	*Hard*	17	1.43	0.34	1.50	0.80	2.20	
	*Durasoft*	16	1.64	0.45	1.60	1.00	2.80	
	*Control*	15	1.58	0.46	1.60	0.90	2.80	
**Ratio opening left**	*Soft*	16	0.41	0.17	0.44	0.10	0.72	0.203
	*Hard*	17	0.40	0.22	0.37	0.15	0.84	
	*Durasoft*	16	0.50	0.17	0.46	0.22	0.84	
	*Control*	15	0.51	0.21	0.48	0.28	0.87	
**Ratio opening right**	*Soft*	16	0.46	0.16	0.49	0.24	0.83	0.062
	*Hard*	17	0.40	0.16	0.38	0.20	0.71	
	*Durasoft*	16	0.54	0.22	0.50	0.17	0.94	
	*Control*	15	0.59	0.22	0.62	0.27	1.04	
**Ratio closing left**	*Soft*	16	0.40	0.16	0.41	0.18	0.88	0.008
	*Hard*	17	0.46	0.19	0.46	0.19	0.97	
	*Durasoft*	16	0.60	0.23	0.53	0.30	1.01	
	*Control*	15	0.55	0.12	0.60	0.36	0.71	
**Ratio closing right**	*Soft*	16	0.48	0.19	0.45	0.22	0.99	0.173
	*Hard*	17	0.50	0.21	0.48	0.19	0.91	
	*Durasoft*	16	0.64	0.27	0.64	0.26	1.35	
	*Control*	15	0.56	0.16	0.55	0.31	0.86	
**PA opening left**	*Soft*	16	0.40	0.47	0.20	0.10	2.00	0.182
	*Hard*	17	0.38	0.35	0.20	0.10	1.10	
	*Durasoft*	16	0.21	0.11	0.20	0.10	0.50	
	*Control*	15	0.20	0.10	0.20	0.10	0.40	
**PA opening right**	*Soft*	16	0.25	0.14	0.20	0.10	0.70	0.060
	*Hard*	17	0.32	0.18	0.30	0.10	0.80	
	*Durasoft*	16	0.24	0.21	0.20	0.10	0.90	
	*Control*	15	0.19	0.07	0.20	0.10	0.30	
**PA closing left**	*Soft*	16	0.34	0.24	0.30	0.10	1.10	0.036
	*Hard*	17	0.26	0.26	0.20	0.10	1.20	
	*Durasoft*	16	0.18	0.09	0.20	0.10	0.40	
	*Control*	15	0.19	0.05	0.20	0.10	0.30	
**PA closing right**	*Soft*	16	0.33	0.25	0.25	0.10	1.10	0.026
	*Hard*	17	0.22	0.10	0.20	0.10	0.50	
	*Durasoft*	16	0.17	0.07	0.20	0.10	0.30	
	*Control*	15	0.18	0.07	0.20	0.10	0.30	
**PF opening left**	*Soft*	16	73.63	48.64	60.00	33.00	208.00	0.892
	*Hard*	17	73.82	58.47	68.00	13.00	259.00	
	*Durasoft*	16	117.00	140.77	54.00	29.00	455.00	
	*Control*	15	97.40	132.49	52.00	25.00	435.00	
**PF opening right**	*Soft*	16	94.06	80.79	74.00	33.00	380.00	0.727
	*Hard*	17	86.71	67.78	60.00	21.00	259.00	
	*Durasoft*	16	130.44	131.13	60.00	37.00	447.00	
	*Control*	15	105.93	132.83	56.00	29.00	451.00	
**PF closing left**	*Soft*	16	70.56	66.07	44.00	21.00	244.00	0.048
	*Hard*	17	80.65	86.20	52.00	25.00	376.00	
	*Durasoft*	16	132.56	130.34	66.00	25.00	408.00	
	*Control*	15	46.47	15.63	48.00	25.00	87.00	
**PF closing right**	*Soft*	16	104.69	116.59	50.00	25.00	435.00	0.396
	*Hard*	17	112.47	136.57	56.00	29.00	478.00	
	*Durasoft*	16	156.19	158.38	77.50	17.00	494.00	
	*Control*	15	53.20	22.26	52.00	25.00	103.00	
**MF opening left**	*Soft*	16	181.38	50.47	183.00	80.00	263.00	0.395
	*Hard*	17	178.88	51.89	166.00	111.00	271.00	
	*Durasoft*	16	203.25	40.56	187.00	142.00	279.00	
	*Control*	15	199.60	58.16	208.00	126.00	279.00	
**MF opening right**	*Soft*	16	197.81	45.13	197.00	91.00	279.00	0.280
	*Hard*	17	186.94	45.66	177.00	103.00	240.00	
	*Durasoft*	16	217.13	49.04	220.00	123.00	291.00	
	*Control*	15	217.47	52.79	232.00	138.00	310.00	
**MF closing left**	*Soft*	16	169.50	48.80	171.00	72.00	287.00	0.008
	*Hard*	17	180.59	49.62	173.00	103.00	298.00	
	*Durasoft*	16	220.63	53.69	212.00	130.00	302.00	
	*Control*	15	214.00	36.19	232.00	142.00	259.00	
**MF closing right**	*Soft*	16	191.31	50.47	185.00	72.00	298.00	0.148
	*Hard*	17	203.94	52.18	220.00	107.00	291.00	
	*Durasoft*	16	230.50	51.22	230.00	138.00	326.00	
	*Control*	15	219.40	37.18	224.00	142.00	271.00	

* According to Kruskal–Wallis test and Bonferroni Correction, results were considered statistically significant for *p* < 0.0063.

**Table 4:** Multiple comparisons for Table 3.

Multiple Comparisons	*p*-Value
**I < 300 opening right**	
**Soft–control**	*p* < 0.001
**Hard–control**	*p* < 0.001

**Table 5:** Comparison among the groups in terms of measurement times (baseline and post-treatment).

Variables	Groups	N	Mean	Std. Deviation	Median	Min	Max	*p*-Value *
**Tİ opening left**	*Soft*	16	+1.03	1.97	+0.50	−0.60	+5.90	0.004
	*Hard*	17	−0.56	4.24	−0.60	−8.10	+11.00	
	*Durasoft*	16	−1.96	2.76	−1.45	−8.30	+1.70	
	*Control*	15	−0.33	0.94	+0.10	−2.80	+0.60	
**Tİ opening right**	*Soft*	16	+0.03	1.71	+0.10	−4.20	+3.70	0.179
	*Hard*	17	−0.54	3.24	+0.40	−9.20	+4.80	
	*Durasoft*	16	−1.03	2.43	−0.90	−7.90	+3.40	
	*Control*	15	−0.54	0.66	−0.60	−1.80	+1.10	
**Tİ closing left**	*Soft*	16	−0.14	1.52	−0.10	−3.00	+2.60	0.432
	*Hard*	17	−0.86	2.30	−0.70	−7.10	+2.80	
	*Durasoft*	16	−0.98	1.55	−0.75	−4.10	+1.20	
	*Control*	15	−0.51	0.96	−0.60	−2.20	+1.40	
**Tİ closing right**	*Soft*	16	−0.24	0.95	+0,05	−2.20	+1.10	0.925
	*Hard*	17	−0.78	1.75	0.00	−5.10	+1.30	
	*Durasoft*	16	−0.53	1.18	−0.75	−2.90	+1.50	
	*Control*	15	−0.41	0.92	−0.20	−1.80	+1.00	
**I < 300 opening left**	*Soft*	16	+1.01	1.88	+0.50	−0.60	+5.80	0.004
	*Hard*	17	−0.03	3.81	−0.70	−8.40	+10.70	
	*Durasoft*	16	−1.78	2.45	−1.35	−7.30	+1.90	
	*Control*	15	−0.22	0.81	−0.40	−1.50	+1.00	
**I < 300 opening right**	*Soft*	16	+0.14	1.87	+0.15	−4.90	+3.50	0.160
	*Hard*	17	−0.37	2.90	+0.10	−8.60	+4.60	
	*Durasoft*	16	−0.88	1.83	−0.85	−4.90	+2.90	
	*Control*	15	−0.45	0.66	−0.30	−1.60	+0.80	
**I < 300 closing left**	*Soft*	16	−0.04	1.39	+0.10	−2.60	+2.60	0.341
	*Hard*	17	−0.60	1.77	−0.50	−4.30	+2.30	
	*Durasoft*	16	−1.01	1.41	−1.05	−3.50	+0.80	
	*Control*	15	−0.51	0.80	−0.50	−1.90	+0.90	
**I < 300 closing right**	*Soft*	16	−0.01	1.02	+0.05	−1.70	+1.50	0.680
	*Hard*	17	−0.59	1.39	−0.10	−3.80	+1.10	
	*Durasoft*	16	−0.53	1.27	−0.55	−3.40	+1.00	
	*Control*	15	−0.35	0.95	−0.20	−2.30	+1.00	
**I > 300 opening left**	*Soft*	16	+0.04	0.42	0.00	−1.00	+0.60	0.279
	*Hard*	17	−0.53	1.07	−0.10	−4.10	+0.40	
	*Durasoft*	16	−0.18	0.46	−0.25	−1.10	+0.50	
	*Control*	15	−0.10	0.59	−0.10	−1.30	+0.80	
**I > 300 opening right**	*Soft*	16	−0.11	0.58	−0.05	−1.50	+0.70	0.992
	*Hard*	17	−0.18	0.65	0.00	−2.40	+0.30	
	*Durasoft*	16	−0.17	0.90	−0.15	−3.00	+1.10	
	*Control*	15	−0.09	0.67	+0.10	−1.00	+1.20	
**I > 300 closing left**	*Soft*	16	−0.17	0.49	−0.20	−1.10	+0.80	0.519
	*Hard*	17	−0.27	0.77	0.00	−2.70	+0.60	
	*Durasoft*	16	+0.03	0.40	−0.15	−0.50	+0.70	
	*Control*	15	+0.01	0.57	+0.10	−0.90	+1.10	
**I > 300 closing right**	*Soft*	16	−0.24	0.57	−0.15	−1.30	+0.60	0.748
	*Hard*	17	−0.19	0.72	0.00	−2.40	+0.80	
	*Durasoft*	16	+0.03	0.54	−0.10	−0.80	+1.20	
	*Control*	15	−0.01	0.78	−0.10	−1.10	+1.40	
**Ratio opening left**	*Soft*	16	−0.08	0.23	−0.04	−0.58	+0.29	0.121
	*Hard*	17	−0.05	0.27	−0.15	−0.46	+0.73	
	*Durasoft*	16	+0.09	0.23	+0.08	−0.38	+0.49	
	*Control*	15	+0.04	0.25	+0.06	−0.40	+0.38	
**Ratio opening right**	*Soft*	16	−0.07	0.30	0.00	−0.68	+0.35	0.578
	*Hard*	17	−0.02	0.14	−0.05	−0.28	+0.20	
	*Durasoft*	16	+0.08	0.27	+0.03	−0.49	+0.61	
	*Control*	15	+0.03	0.32	+0.04	−0.42	+0.76	
**Ratio closing left**	*Soft*	16	−0.10	0.27	−0.06	−0.78	+0.38	0.096
	*Hard*	17	−0.01	0.26	−0.03	−0.47	+0.75	
	*Durasoft*	16	+0.13	0.27	+0.15	−0.25	+0.66	
	*Control*	15	+0.06	0.26	+0.12	−0.43	+0.41	
**Ratio closing right**	*Soft*	16	−0.09	0.31	+0.02	−0.77	+0.37	0.719
	*Hard*	17	+0.04	0.23	−0.03	−0.20	+0.69	
	*Durasoft*	16	+0.08	0.35	+0.06	−0.42	+0.80	
	*Control*	15	−0.01	0.32	−0.07	−0.53	+0.64	
**PA opening left**	*Soft*	16	+0.12	0.27	+0.10	−0.20	+1.00	0.007
	*Hard*	17	−0.02	0.45	0.00	−1.50	+0.80	
	*Durasoft*	16	−0.14	0.20	−0.10	−0.60	+0.10	
	*Control*	15	−0.01	0.07	0.00	−0.10	+0.10	
**PA opening right**	*Soft*	16	+0.01	0.20	0.00	−0.50	+0.50	0.179
	*Hard*	17	−0.04	0.26	0.00	−0.70	+0.30	
	*Durasoft*	16	−0.09	0.15	−0.10	−0.40	+0.20	
	*Control*	15	−0.02	0.06	0.00	−0.10	+0.10	
**PA closing left**	*Soft*	16	+0.04	0.22	0.00	−0.40	+0.50	0.167
	*Hard*	17	−0.05	0.21	−0.10	−0.30	+0.50	
	*Durasoft*	16	−0.11	0.18	−0.10	−0.50	+0.10	
	*Control*	15	−0.05	0.06	0.00	−0.20	0.00	
**PA closing right**	*Soft*	16	+0.03	0.17	0.00	−0.20	+0.50	0.080
	*Hard*	17	−0.06	0.14	−0.10	−0.30	+0.10	
	*Durasoft*	16	−0.13	0.16	−0.10	−0.60	+0.10	
	*Control*	15	−0.05	0.10	−0.10	−0.20	+0.10	
**PF opening left**	*Soft*	16	−55.81	146.84	0.00	−414.00	+105.00	0.903
	*Hard*	17	−48.59	131.68	−15.00	−324.00	+230.00	
	*Durasoft*	16	−4.38	176.41	−8.00	−414.00	+387.00	
	*Control*	15	+1.67	114.85	−16.00	−191.00	+363.00	
**PF opening right**	*Soft*	16	−49.75	121.24	−11.50	−309.00	+164.00	0.774
	*Hard*	17	−21.59	114.66	+4.00	−316.00	+199.00	
	*Durasoft*	16	+12.69	165.78	−5.50	−301.00	+379.00	
	*Control*	15	+8.67	103.58	+8.00	−191.00	+321.00	
**PF closing left**	*Soft*	16	−15.19	139.26	+4.00	−363.00	+192.00	0.540
	*Hard*	17	−20.53	154.88	+4.00	−383.00	+316.00	
	*Durasoft*	16	−17.50	185.71	+2.00	−410.00	+328.00	
	*Control*	15	−53.27	122.58	−11.00	−403.00	+19.00	
**PF closing right**	*Soft*	16	−3.25	131.29	+7.00	−414.00	+160.00	0.547
	*Hard*	17	+34.41	162.41	+16.00	−278.00	+379.00	
	*Durasoft*	16	−9.44	237.11	+7.50	−422.00	+461.00	
	*Control*	15	−44.80	128.07	0.00	−407.00	+42.00	
**MF opening left**	*Soft*	16	−14.81	57.53	−21.00	−105.00	+90.00	0.239
	*Hard*	17	−17.53	72.03	−31.00	−110.00	+203.00	
	*Durasoft*	16	+16.75	59.06	+13.00	−82.00	+129.00	
	*Control*	15	+4.07	68.47	+16.00	−117.00	+117.00	
**MF opening right**	*Soft*	16	−15.63	73.23	−23.00	−125.00	+109.00	0.346
	*Hard*	17	−3.65	45.32	−12.00	−74.00	+109.00	
	*Durasoft*	16	+23.50	56.13	+23,50	−78.00	+109.00	
	*Control*	15	−0.80	74.60	−4.00	−102.00	+156.00	
**MF closing left**	*Soft*	16	−23.75	71.03	−31.00	−133.00	+82.00	0.169
	*Hard*	17	−7.88	76.17	+4.00	−137.00	+168.00	
	*Durasoft*	16	+33.75	74.96	+35.00	−102.00	+179.00	
	*Control*	15	+18.07	68.78	+27.00	−117.00	+125.00	
**MF closing right**	*Soft*	16	−20.88	66.59	−6.00	−129.00	+94.00	0.379
	*Hard*	17	+10.65	62.00	−4.00	−74.00	+157.00	
	*Durasoft*	16	+27.19	74.19	+27,50	−82.00	+168.00	
	*Control*	15	+2.13	72.59	−15.00	−137.00	+137.00	

* According to Kruskal–Wallis test and Bonferroni Correction, results were considered statistically significant for *p* < 0.0125.

## Data Availability

All data generated or analyzed during this study are included in this article. Further enquiries can be directed toward the corresponding author.

## Supplementary Materials

The following supporting information can be downloaded at https://www.mdpi.com/article/10.3390/medicina61061083/s1, File S1: The exclusion criteria and approximate distribution of excluded individuals; File S2: Pre-treatment and post-treatment anamnesis form results; File S3: Pre-treatment and post-treatment clinical examination findings; File S4: Graphs of the amount of change for JVA parameters according to time, movement and right-left joints.
