# Supplementary material for: Effects of Different Occlusal Splints on Joint Vibrations in Bruxers
Source: Medicina (Kaunas). 2025 Jun 12;61(6):1083. doi: 10.3390/medicina61061083 (PMC12195145; doi:10.3390/medicina61061083)
Supplement: Supplementary file 1 [file medicina-61-01083-s001.zip › Supplement file S1.pdf]

Table . The exclusion criteria and approximate distribution of excluded individuals

A. Absence of reported symptoms (e.g., no clenching/grinding): n = 107
B. Presence of temporomandibular joint disorders (TMD) or pain-related symptoms (beyond early bruxism stage): n = 25
C. Medical or psychiatric disorders affecting masticatory muscle function (e.g., neurological conditions, antidepressant use): n = 19
D. Failure to provide informed consent or withdrawal before group assignment: n = 23
