# Supplementary material for: Effects of Different Occlusal Splints on Joint Vibrations in Bruxers
Source: Medicina (Kaunas). 2025 Jun 12;61(6):1083. doi: 10.3390/medicina61061083 (PMC12195145; doi:10.3390/medicina61061083)
Supplement: Supplementary file 1 [file medicina-61-01083-s001.zip › Supplement file S4.pdf]

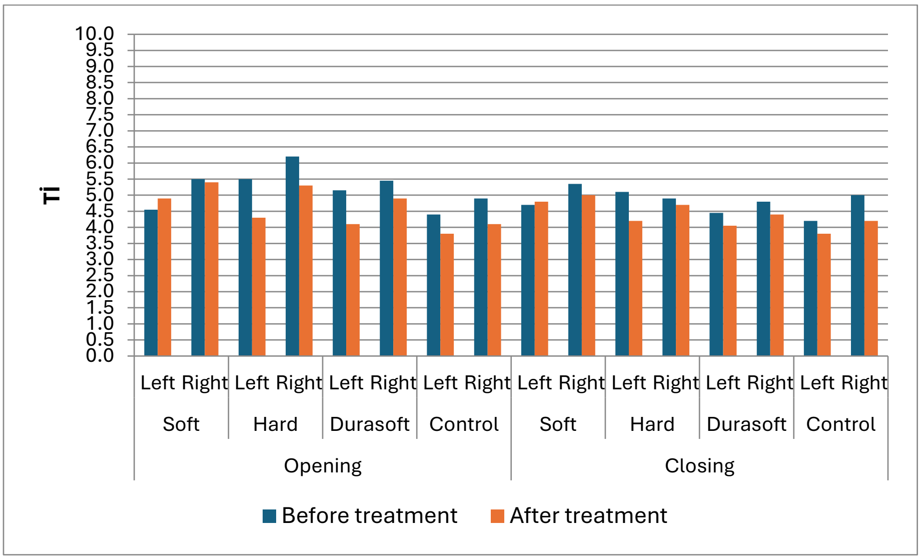

Graph 1. The amount of change for Ti according to time, movement and right-left joints.

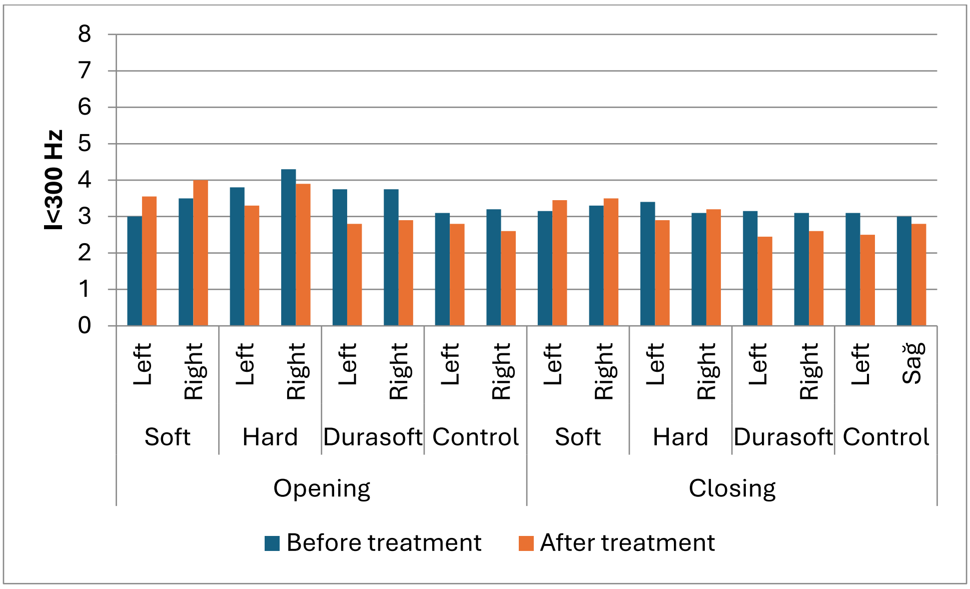

Graph 2. The amount of change for İ<300 Hz according to time, movement and right-left joints.

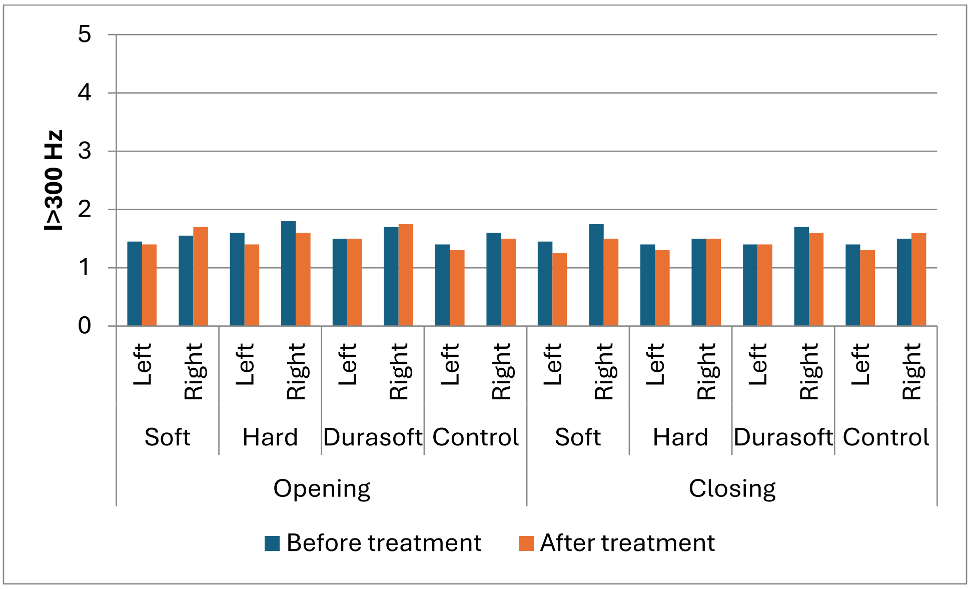

Graph 3. The amount of change for i>300 Hz according to time, movement and right-left joints.

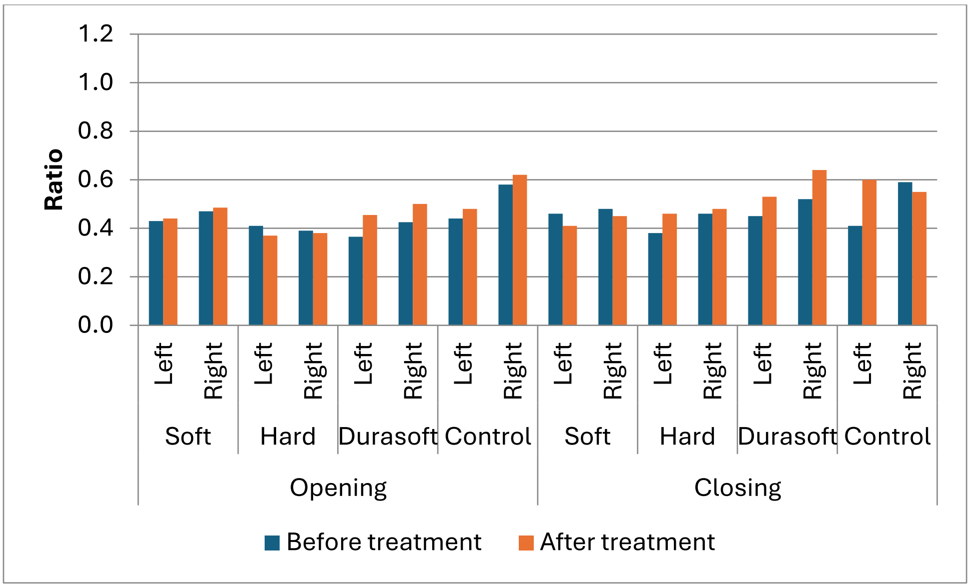

Graph 4. The amount of change for Ratio according to time, movement and right-left joints.

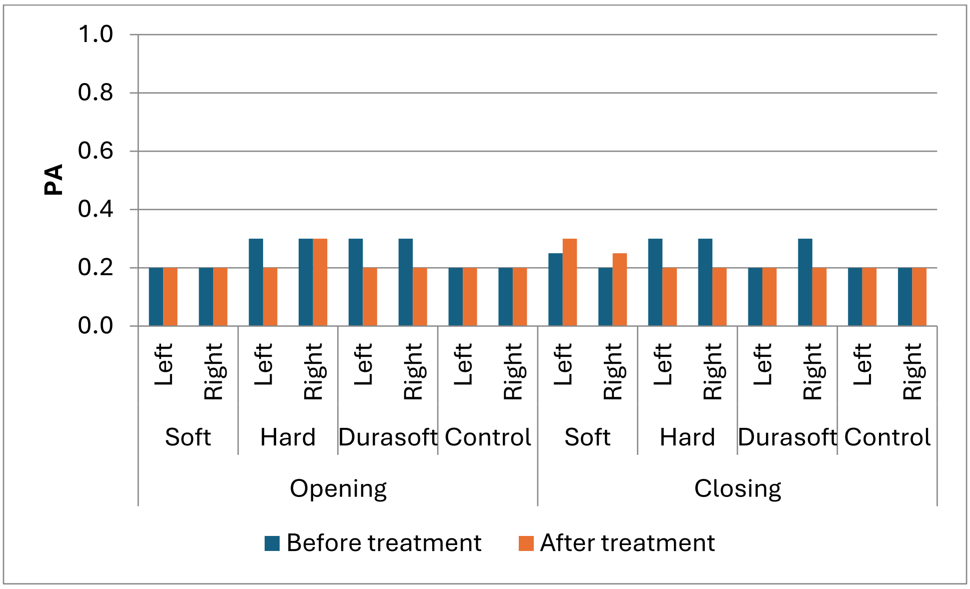

Graph 5. The amount of change for PA according to time, movement and right-left joints.

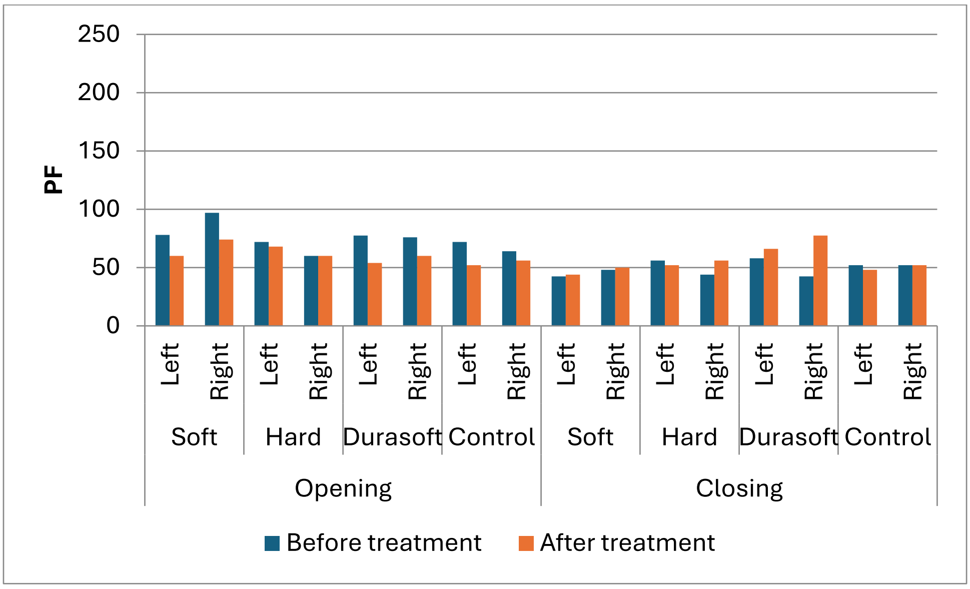

Graph 6. The amount of change for PF according to time, movement and right-left joints.

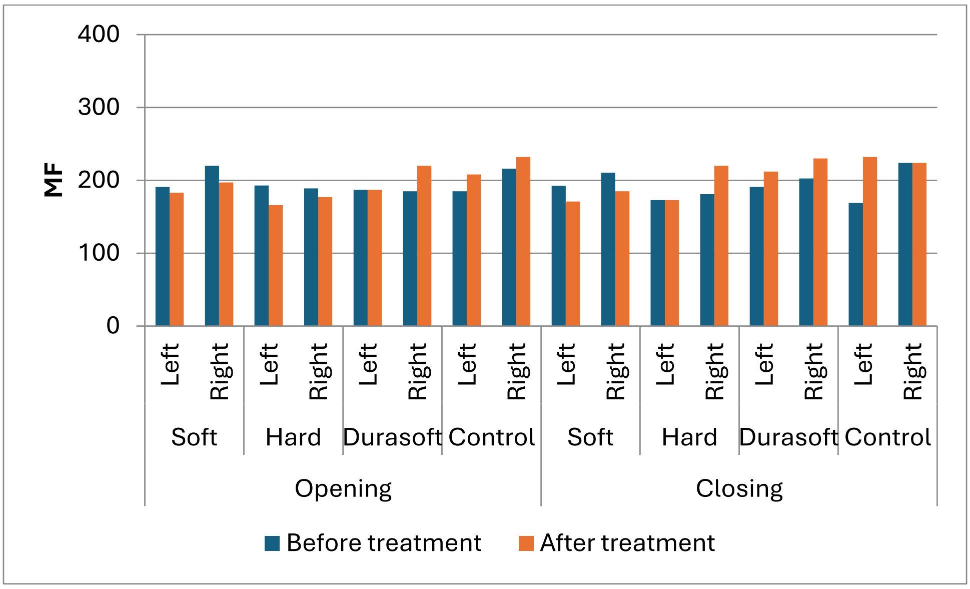

Graph 7. The amount of change for MF according to time, movement and right-left joints.
